# Two-dimensional NMR data of a series of methylcellulose with different degrees of substitution

**DOI:** 10.1016/j.dib.2018.04.009

**Published:** 2018-04-05

**Authors:** Hiroyuki Kono

**Affiliations:** Division of Applied Chemistry and Biochemistry, National Institute of Technology, Tomakomai College, Nishikioka 443, Tomakomai, Hokkaido 0591275, Japan

**Keywords:** NMR, Methylcellulose, Cellulose ether, Spectral data

## Abstract

This article contains two-dimensional (2D) NMR experimental data for a series of methylcellulose (MC) with different substitution degrees (DS), obtained by the Bruker BioSpin 500 MHz NMR spectrometer (Germany). The data facilitated the ^1^H and ^13^C chemical shifts of eight anhydroglucose units (AGUs) comprising MC chains–unsubstituted, 2-mono-, 3-mono-, 6-mono-, 2,3-di-, 2,6-di-, 3,6-di-, and 2,3,6-tri-substituted AGUs. Data include analyzed the 2D NMR spectra of the MC samples, which are related to the subject of an article in *Carbohydrate Polymers*, entitled “NMR characterization of methylcellulose: Chemical shift assignment and mole fraction of monomers in the polymer chains” (Kon et al., 2017) [1]. These data can be very helpful to assign the ^1^H and ^13^C chemical shifts of the other cellulose derivatives, especially cellulose ethers.

**Specifications table**

**Table:** 

Subject area	*Chemistry*
More specific subject area	*Structural analysis*
Type of data	*NMR spectra*
How data was acquired	*NMR, Bruker BioSpin AVIII* 500* *MHz *spectrometer equipped with a Bruker BioSpin z-gradient dual-resonance BBFO probe (*5* *mm*).*
Data format	*Analyzed*
Experimental factors	*About 35 *mg *of each sample dissolved in 700 μL of 99.9% dimethylsulfoxide (DMSO)-d*_*6*_ containing 0.03% tetramethylsilane (TMS).
Experimental features	*All NMR experiments were performed at 363 *K*.*
Data source location	*National Institute of Technology, Tomakomai College, Nishikioka 443, Tomakomai, Hokkaido 0591275, Japan*
Data accessibility	*Data are with this article.*

**Value of the data**

• The following data detail NMR characterization of a MC samples with different DS.
• The NMR data can be helpful to assign the chemical shifts of AGUs comprising other cellulose derivatives.
• NMR parameters used for the obtained data can be useful for structural characterization of complex polysaccharides.

## 1 Data

**Fig. 1:**
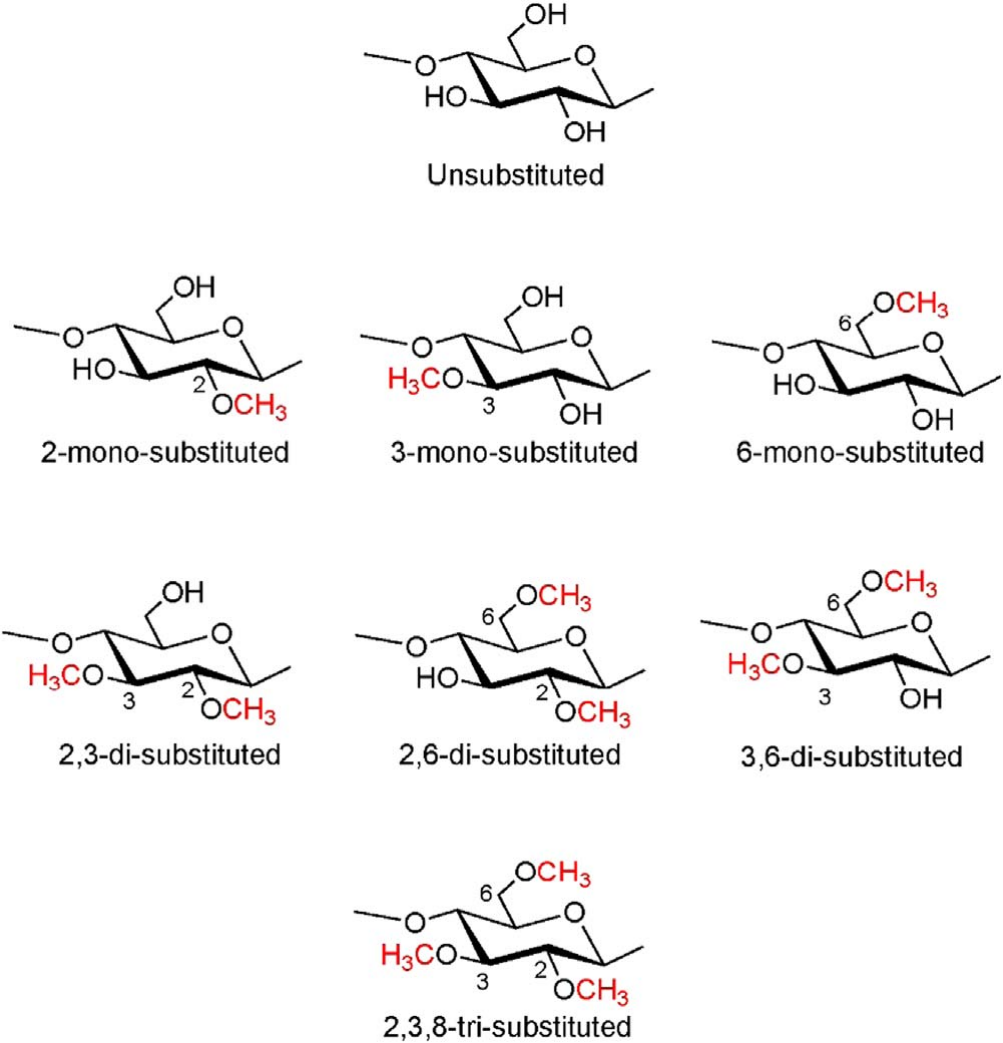
Eight anhydroglucose units (AGUs) comprising methylcellulose (MC) chains.

Cellulose is a linear 1,4-β-D-glucan with three hydroxyl groups per anhydroglucose units (AGU). Each AGU contains three hydroxyl groups at the 2, 3, and 6 positions. In the case of methylcellulose (MC), substitution can occur at theses hydroxyl groups, resulting in the formation of 8 different AGUs in the structure except for total degree of substitution (DS) of 3, namely, unsubstituted, 2-mono-, 3-mono-, 6-mono-, 2,3-di-, 2,6-di-, 3,6-di-, and 2,3,6-tri-substituted AGUs (Fig. 1). The complexity of the chemical structure of MC samples, and thus their properties, conformation, and dynamics is related to the substituent distribution as well as the total DS of the derivatives [1], [2], [3].

**Fig. 2:**
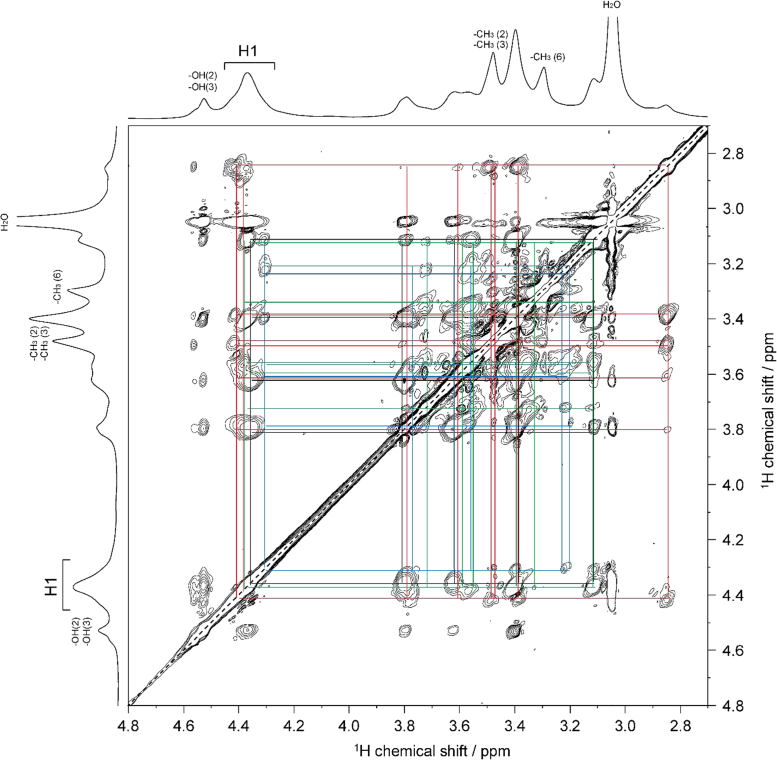
2D ^1^H–^1^H TOCSY spectrum of MC**1** (DS=0.66) in DMSO-*d*_*6*_ at 363 K. ^1^H NMR spectrum of MC**1** is shown in horizontal and vertical axis. Assignment for methyl groups substituted at the 2-, 3- and 6-positions of cellulose denoted as –CH_3_(2), –CH_3_(3), and –CH_3_(6), respectively, are indicated in the ^1^H spectrum. Through-bond ^1^H–^1^H spin coupling networks of unsubstituted, 2-mono-, 3-mono-, 6-mono-substituted AGUs are denoted by black, red, blue, and green lines in the TOCSY spectrum, respectively.

**Fig. 3:**
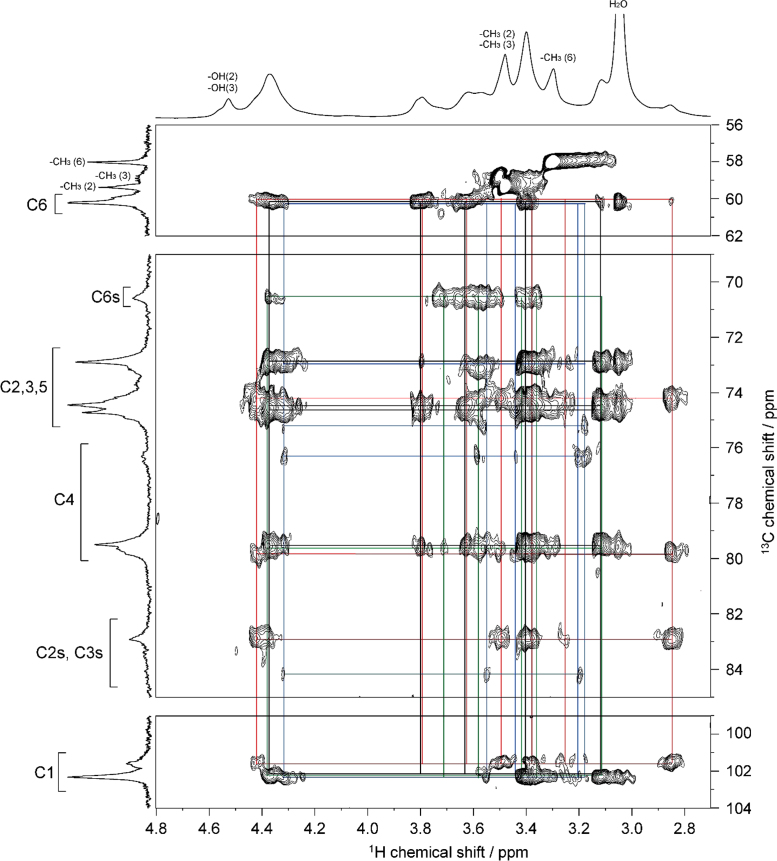
2D ^1^H–^13^C HSQC-TOCSY spectrum of MC**1** (DS=0.66) in DMSO-*d*_*6*_ at 363 K. ^1^H and ^13^C NMR spectra of MC**1** are shown in horizontal and vertical axis, respectively. C2s, C3s, and C6s indicate the C2, C3, and C6 resonances, respectively, where the hydroxyl groups are substituted by methyl groups. Assignment for methyl groups substituted at the 2-, 3- and 6-positions of cellulose, denoted as –CH_3_(2), –CH_3_(3), and –CH_3_(6), respectively, are indicated in the ^1^H and ^13^C spectra. Through-bond ^1^H–^13^C spin coupling networks of unsubstituted, 2-mono-, 3-mono-, 6-mono-substituted AGUs are denoted by black, red, blue, and green lines in the HSQC-TOCSY spectrum, respectively.

**Fig. 4:**
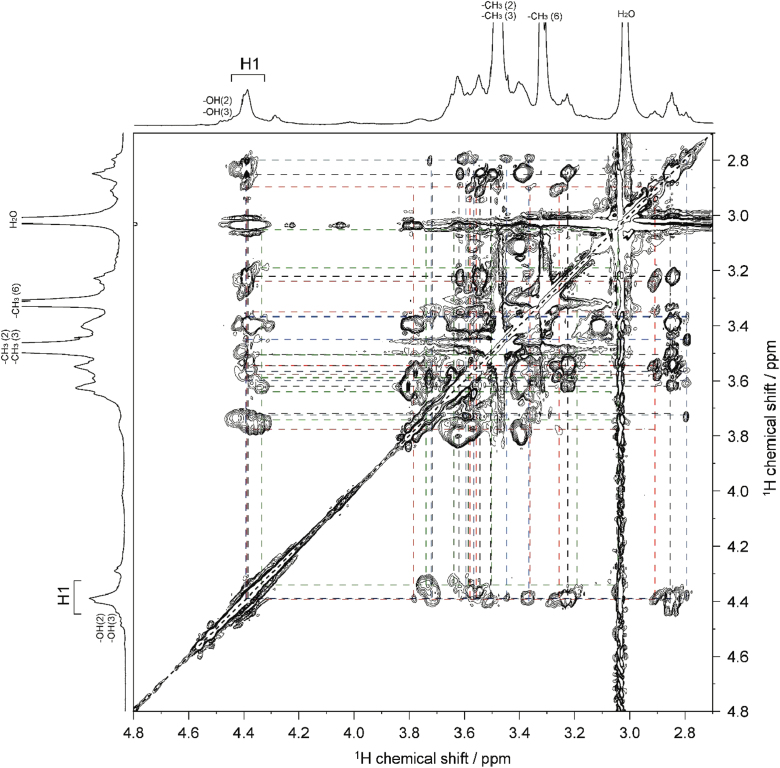
2D ^1^H–^1^H TOCSY spectrum of MC**4** (DS=2.38) in DMSO-*d*_*6*_ at 363 K. ^1^H NMR spectrum of MC**4** is shown in horizontal and vertical axis. Assignment for methyl groups substituted at the 2-, 3- and 6-positions of cellulose denoted as –CH_3_(2), –CH_3_(3), and –CH_3_(6), respectively, are indicated in the ^1^H spectrum. Through-bond ^1^H–^1^H spin coupling networks of 2,3,6-tri-, 2,3-di-, 2,6-di-, 3,6-di-substituted AGUs are denoted by dotted black, red, blue, and green lines in the TOCSY spectrum, respectively.

**Fig. 5:**
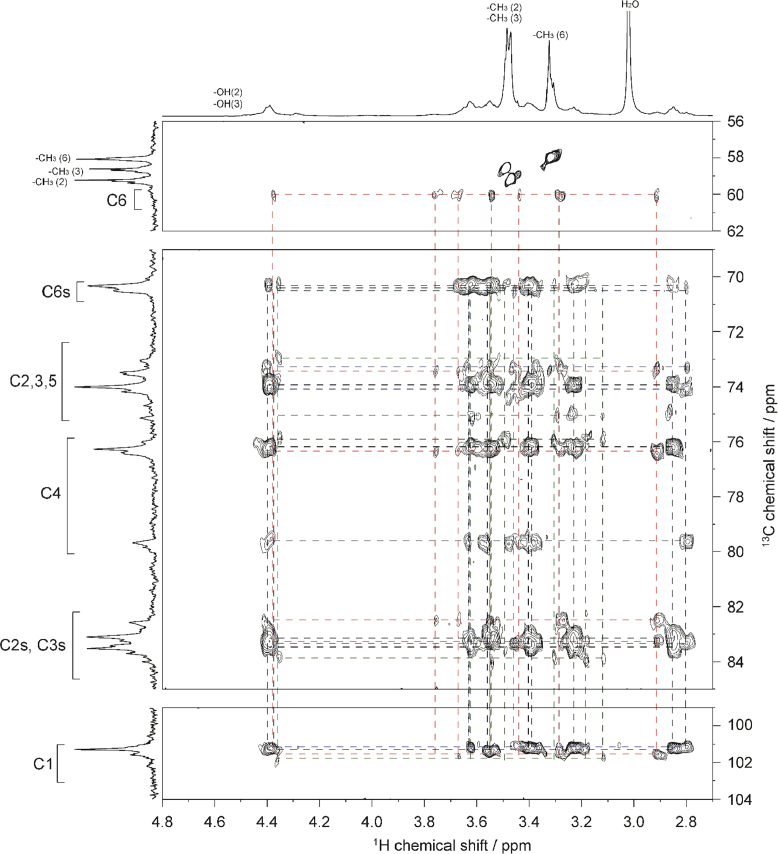
2D ^1^H–^13^C HSQC-TOCSY spectrum of MC**4** (DS=2.38) in DMSO-*d*_*6*_ at 363 K. ^1^H and ^13^C NMR spectra of MC**4** are shown in horizontal and vertical axis, respectively. C2s, C3s, and C6s indicate the C2, C3, and C6 resonances, respectively, where the hydroxyl groups are substituted by methyl groups. Assignment for methyl groups substituted at the 2-, 3- and 6-positions of cellulose, denoted as –CH_3_(2), –CH_3_(3), and –CH_3_(6), respectively, are indicated in the ^1^H and ^13^C spectra. Through-bond ^1^H–^13^C spin coupling networks of 2,3,6-tri-, 2,3-di-, 2,6-di-, 3,6-di-substituted AGUs are denoted by dotted black, red, blue, and green lines in the HSQC-TOCSY spectrum, respectively.

**Fig. 6:**
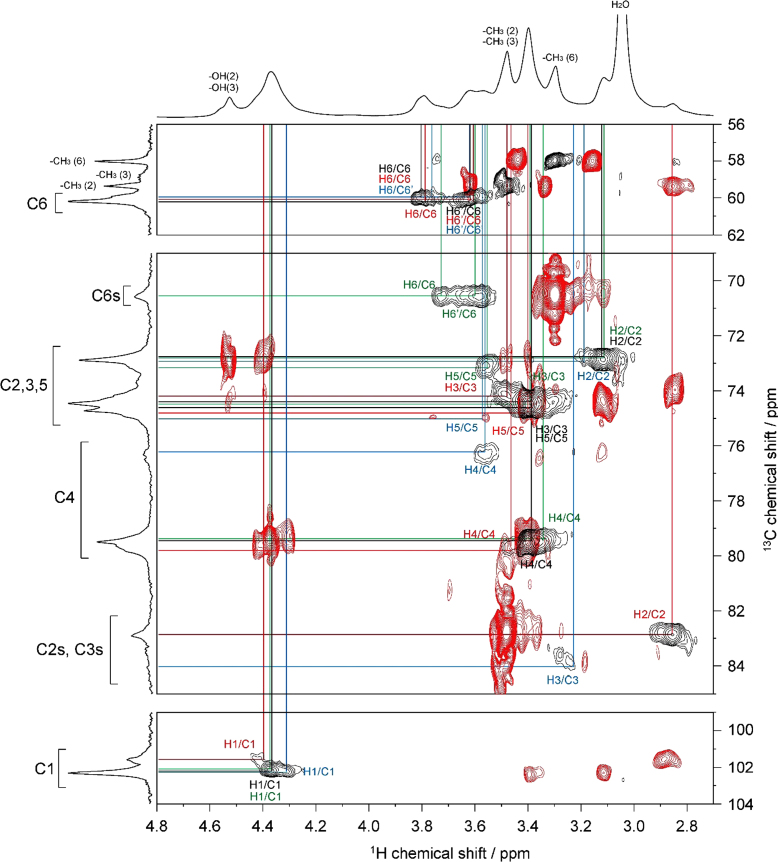
2D ^1^H–^13^C HSQC (black lines) and HMBC (red lines) spectra of MC**1** (DS=0.66) in DMSO-*d*_*6*_ at 363 K. ^1^H and ^13^C NMR spectra of MC**1** are shown in horizontal and vertical axis, respectively. C2s, C3s, and C6s indicate the C2, C3, and C6 resonances, respectively, where the hydroxyl groups are substituted by methyl groups. Assignment for methyl groups substituted at the 2-, 3- and 6-positions of cellulose, denoted as –CH_3_(2), –CH_3_(3), and –CH_3_(6), respectively, are indicated in the ^1^H and ^13^C spectra. Directly-coupled ^1^H–^13^C spin couplings of unsubstituted, 2-mono-, 3-mono-, 6-mono-substituted AGUs are denoted by black, red, blue, and green lines in the spectrum, respectively.

**Fig. 7:**
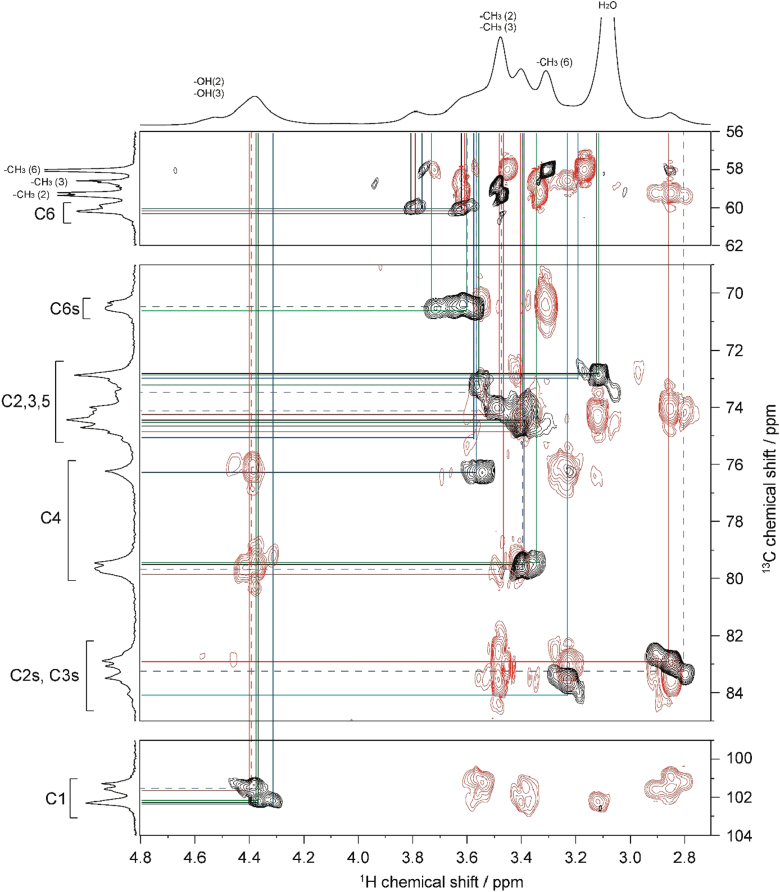
2D ^1^H–^13^C HSQC (black lines) and HMBC (red lines) spectra of MC**2** (DS=1.27) in DMSO-*d*_*6*_ at 363 K. ^1^H and ^13^C NMR spectra of MC**2** are shown in horizontal and vertical axis, respectively. C2s, C3s, and C6s indicate the C2, C3, and C6 resonances, respectively, where the hydroxyl groups are substituted by methyl groups. Assignment for methyl groups substituted at the 2-, 3- and 6-positions of cellulose, denoted as –CH_3_(2), –CH_3_(3), and –CH_3_(6), respectively, are indicated in the ^1^H and ^13^C spectra. Directly-coupled ^1^H–^13^C spin couplings of unsubstituted, 2-mono-, 3-mono-, 6-mono-, and 2,6-di-substituted, AGUs are denoted by black, red, blue, green, and dotted blue lines in the spectrum, respectively.

**Fig. 8:**
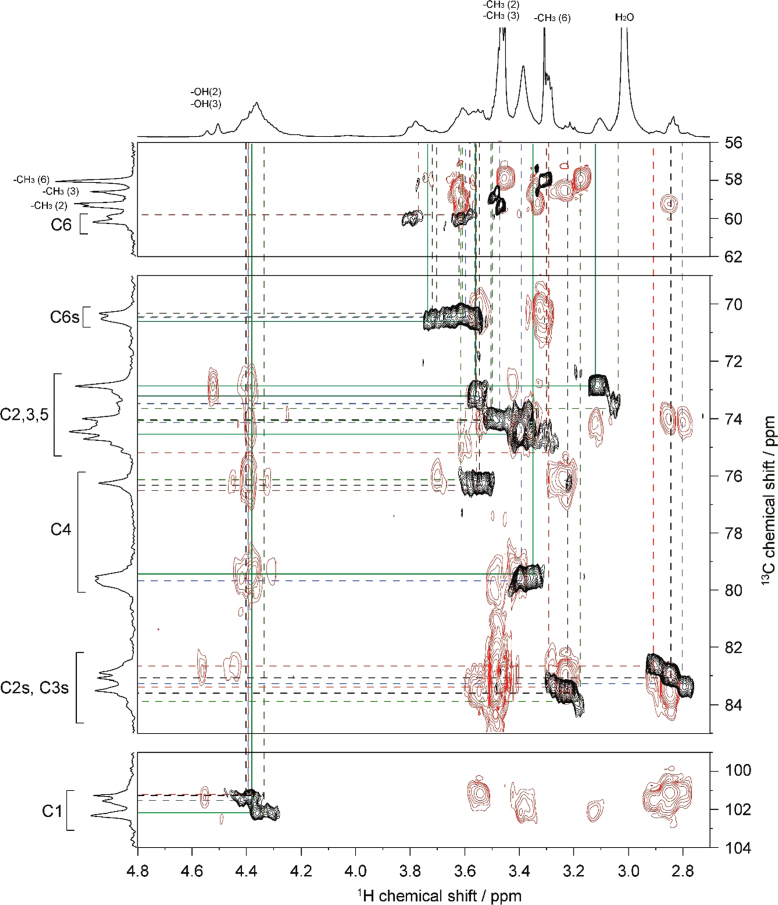
2D ^1^H–^13^C HSQC (black lines) and HMBC (red lines) spectra of MC**3** (DS=1.64) in DMSO-*d*_*6*_ at 363 K. ^1^H and ^13^C NMR spectra of MC**3** are shown in horizontal and vertical axis, respectively. C2s, C3s, and C6s indicate the C2, C3, and C6 resonances, respectively, where the hydroxyl groups are substituted by methyl groups. Assignment for methyl groups substituted at the 2-, 3- and 6-positions of cellulose, denoted as –CH_3_(2), –CH_3_(3), and –CH_3_(6), respectively, are indicated in the ^1^H and ^13^C spectra. Directly-coupled ^1^H–^13^C spin couplings of 6-mono-, 2,3-di-, 2,6-di-, 3,6-di-, and 2,3,6-tri-substituted, AGUs are denoted by green, dotted red, dotted blue, dotted green, and dotted black lines in the spectrum, respectively.

**Fig. 9:**
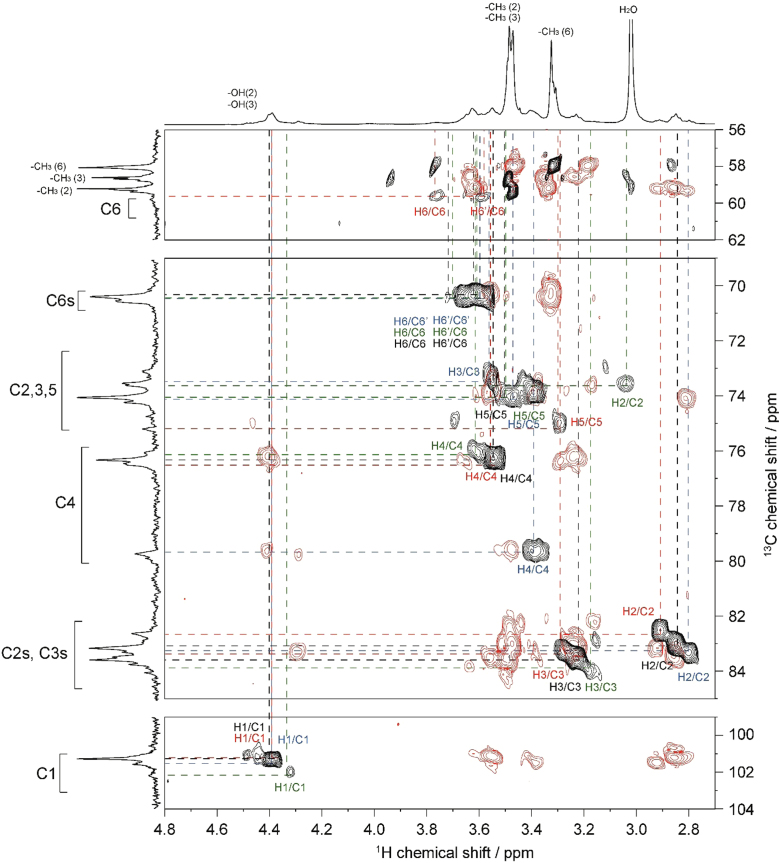
2D ^1^H–^13^C HSQC (black lines) and HMBC (red lines) spectra of MC**4** (DS=2.38) in DMSO-*d*_*6*_ at 363 K. ^1^H and ^13^C NMR spectra of MC**3** are shown in horizontal and vertical axis, respectively. C2s, C3s, and C6s indicate the C2, C3, and C6 resonances, respectively, where the hydroxyl groups are substituted by methyl groups. Assignment for methyl groups substituted at the 2-, 3- and 6-positions of cellulose, denoted as –CH_3_(2), –CH_3_(3), and –CH_3_(6), respectively, are indicated in the ^1^H and ^13^C spectra. Directly-coupled ^1^H–^13^C spin couplings of 6-mono-, 2,3-di-, 2,6-di-, 3,6-di-, and 2,3,6-tri-substituted, AGUs are denoted by green, dotted red, dotted blue, dotted green, and dotted black lines in the spectrum, respectively.

The presented data include 2D NMR spectra of four MC samples (MC **1**–**4**) whose DS were 0.66, 1.27, 1.64, and 2.38, respectively. 2D ^1^H–^1^H total correlation spectroscopy (TOCSY) and 2D ^1^H–^13^C heteronuclear single quantum coherence (HSQC)-TOCSY spectra of MC **1** are shown in Fig. 2, Fig. 3, respectively. 2D ^1^H–^1^H TOCSY and 2D ^1^H–^13^C heteronuclear single quantum coherence (HSQC)-TOCSY spectra of MC **4** are shown in Fig. 4, Fig. 5, respectively. 2D ^1^H–^13^C HSQC and 2D ^1^H–^13^C heteronuclear multiple-bond correlation (HMBC) of MC **1**–**4** are shown in Fig. 6, Fig. 7, Fig. 8, Fig. 9.

## 2 Experimental design, materials and methods

The experiment's planning, design, and data processing correspond to the protocol given in Ref. [1].

### 2.1 Samples

MC samples were prepared to a previously reported method [1].

### 2.2 Description of the NMR experiments

Each MC (about 35 mg) was dissolved in 700 μL of DMSO-*d*_6_ (99.9% isotropic purity, Sigma-Aldrich). All NMR spectra were recorded on a Bruker BioSpin AVIII 500 MHz spectrometer equipped with a Bruker BioSpin *z*-gradient dual-resonance BBFO probe (5 mm) at 363 K. 2D ^1^H–^1^H TOCSY data were acquired on a 2048×256-point matrix for the full spectrum, with 64 scans per increment, and the TOCSY spin-locking time was set to 100 ms. 2D ^1^H–^13^C HSQC-TOCSY data were acquired on a 1024×128-point matrix for the full spectrum, with 128 scans per increment. The TOCSY spin-locking time and interpulse delay which corresponded to 1/4 *J*_*CH*_ in the HSQC-TOCSY experiment was set to 100 ms and 3.44 ms, respectively. 2D ^1^H–^13^C HSQC data were acquired on a 1024×128-point matrix for the full spectrum, with 128 scans per increment, and the interpulse delay which corresponded to 1/4 *J*_*CH*_ was set to 3.44 ms. 2D ^1^H–^13^C HMBC data were acquired on a 2048×256-point matrix for the full spectrum, with 128 scans per increment, and the delay time for the evolution was set to 62.5 ms. The repetition time of each 2D NMR experiment was 2 s. For the TOCSY, HSQC-TOCSY, and HSQC data, a sine-squared window function was applied along both dimensions before the Fourier transform. The HMBC data were linearly predicted to 256 points along the F1 dimension and zero-filled to give 2048×512 points. A sine-bell window function was applied along both dimensions of the HMBC data before the Fourier transform. ^1^H and ^13^C chemical shifts were calibrated using TMS as an internal standard (0 ppm).
